# Supplementary material for: Tolerability of Diquas LX on tear film and meibomian glands findings in a real clinical scenario
Source: PLoS One. 2024 Sep 26;19(9):e0305020. doi: 10.1371/journal.pone.0305020 (PMC11426461; doi:10.1371/journal.pone.0305020)
Supplement: S1 Table — (PDF) [file pone.0305020.s001.pdf]

ID	DQS/DQS-LX group	Age	Sex	Allergic conjunctivitis	Collagen Disease	Sjögren syndrome	Rheumatoid arthritis	SLK	Cataract Surgery
1	DQS	35	M	Allergic conjunctivitis					
2	DQS	71	F	Allergic conjunctivitis					Cataract Surgery
3	DQS	58	F	Allergic conjunctivitis					
4	DQS	72	F	Allergic conjunctivitis					
5	DQS	75	F	Allergic conjunctivitis					
6	DQS	70	F						Cataract Surgery
7	DQS	56	F						
8	DQS	73	F	Allergic conjunctivitis					
9	DQS	75	F	Allergic conjunctivitis					
10	DQS	47	F	Allergic conjunctivitis					
11	DQS	61	F						
12	DQS	60	F	Allergic conjunctivitis	Collagen Disease	Sjögren syndrome			
13	DQS	59	M	Allergic conjunctivitis					
14	DQS	56	M						
15	DQS	70	F		Collagen Disease	Sjögren syndrome			
16	DQS	87	F	Allergic conjunctivitis					Cataract Surgery
17	DQS-LX	68	F						Cataract Surgery
18	DQS-LX	63	F						
19	DQS-LX	39	M						
20	DQS-LX	50	F						
21	DQS-LX	48	F	Allergic conjunctivitis					
22	DQS-LX	65	F		Collagen Disease	Sjögren syndrome			
23	DQS-LX	50	F						
24	DQS-LX	62	F		Collagen Disease		Rheumatoid arthritis		
25	DQS-LX	58	F						
26	DQS-LX	44	F	Allergic conjunctivitis					
27	DQS-LX	50	F	Allergic conjunctivitis					
28	DQS-LX	63	F		Collagen Disease	Sjögren syndrome			
29	DQS-LX	48	F	Allergic conjunctivitis					
30	DQS-LX	57	M						Cataract Surgery
31	DQS-LX	65	M					SLK	
32	DQS-LX	58	F						
33	DQS-LX	60	F	Allergic conjunctivitis				SLK	
34	DQS-LX	77	F						Cataract Surgery
35	DQS-LX	64	F						
36	DQS-LX	80	M						Cataract Surgery
37	DQS-LX	46	F						
38	DQS-LX	65	F						
39	DQS-LX	57	F	Allergic conjunctivitis					
40	DQS-LX	64	F						
41	DQS-LX	73	F						
42	DQS-LX	83	F	Allergic conjunctivitis	Collagen Disease	Sjögren syndrome			
43	DQS-LX	54	F	Allergic conjunctivitis	Collagen Disease	Sjögren syndrome			
44	DQS-LX	64	M						
45	DQS-LX	74	F	Allergic conjunctivitis	Collagen Disease	Sjögren syndrome			
46	DQS-LX	55	M	Allergic conjunctivitis				SLK	
47	DQS-LX	83	F		Collagen Disease		Rheumatoid arthritis		
48	DQS-LX	68	F	Allergic conjunctivitis	Collagen Disease		Rheumatoid arthritis		
