# Supplementary material for: Tolerability of Diquas LX on tear film and meibomian glands findings in a real clinical scenario
Source: PLoS One. 2024 Sep 26;19(9):e0305020. doi: 10.1371/journal.pone.0305020 (PMC11426461; doi:10.1371/journal.pone.0305020)
Supplement: S2 Table — (PDF) [file pone.0305020.s002.pdf]

ID	DQS/DQS-LX group	Eye drops for dry eye	Rebamipide UD	Sodium hyaluronate 0.1%	Sodium hyaluronate Mini 0.3%
1	DQS				
2	DQS				
3	DQS				
4	DQS				
5	DQS				
6	DQS				
7	DQS				
8	DQS	Eye drops for dry eye	Rebamipide UD		
9	DQS				
10	DQS				
11	DQS				
12	DQS				
13	DQS	Eye drops for dry eye	Rebamipide UD		
14	DQS				
15	DQS				
16	DQS				
17	DQS-LX	Eye drops for dry eye	Rebamipide UD		
18	DQS-LX				
19	DQS-LX				
20	DQS-LX				
21	DQS-LX				
22	DQS-LX				
23	DQS-LX				
24	DQS-LX	Eye drops for dry eye	Rebamipide UD		
25	DQS-LX				
26	DQS-LX				
27	DQS-LX				
28	DQS-LX	Eye drops for dry eye	Rebamipide UD		Sodium hyaluronate Mini 0.3%
29	DQS-LX	Eye drops for dry eye	Rebamipide UD		
30	DQS-LX				
31	DQS-LX				
32	DQS-LX				
33	DQS-LX	Eye drops for dry eye	Rebamipide UD		
34	DQS-LX				
35	DQS-LX	Eye drops for dry eye		Sodium hyaluronate 0.1%	
36	DQS-LX	Eye drops for dry eye		Sodium hyaluronate 0.1%	
37	DQS-LX				
38	DQS-LX	Eye drops for dry eye		Sodium hyaluronate 0.1%	
39	DQS-LX				
40	DQS-LX				
41	DQS-LX				
42	DQS-LX				
43	DQS-LX	Eye drops for dry eye	Rebamipide UD		
44	DQS-LX				
45	DQS-LX				
46	DQS-LX				
47	DQS-LX				
48	DQS-LX	Eye drops for dry eye	Rebamipide UD		

ID	DQS/DQS-LX group	Anti-allergic eye drops	Epinastine 0.05%	Epinastine 0.1%	Olopatadine	Azithromycin	Fluorometholone 0.1%	IPL
1	DQS	Anti-allergic eye drops		Epinastine 0.1%			Fluorometholone 0.1%	
2	DQS	Anti-allergic eye drops		Epinastine 0.1%				
3	DQS	Anti-allergic eye drops		Epinastine 0.1%				
4	DQS	Anti-allergic eye drops		Epinastine 0.1%			Fluorometholone 0.1%	
5	DQS	Anti-allergic eye drops		Epinastine 0.1%				
6	DQS						Fluorometholone 0.1%	IPL
7	DQS							
8	DQS	Anti-allergic eye drops			Olopatadine			
9	DQS	Anti-allergic eye drops		Epinastine 0.1%			Fluorometholone 0.1%	
10	DQS	Anti-allergic eye drops		Epinastine 0.1%				
11	DQS							
12	DQS	Anti-allergic eye drops		Epinastine 0.1%				
13	DQS	Anti-allergic eye drops	Epinastine 0.05%					IPL
14	DQS							
15	DQS							
16	DQS	Anti-allergic eye drops		Epinastine 0.1%			Fluorometholone 0.1%	IPL
17	DQS-LX							IPL
18	DQS-LX						Fluorometholone 0.1%	IPL
19	DQS-LX						Fluorometholone 0.1%	
20	DQS-LX					Azithromycin		
21	DQS-LX	Anti-allergic eye drops		Epinastine 0.1%				IPL
22	DQS-LX					Azithromycin	Fluorometholone 0.1%	IPL
23	DQS-LX					Azithromycin		
24	DQS-LX					Azithromycin	Fluorometholone 0.1%	IPL
25	DQS-LX						Fluorometholone 0.1%	IPL
26	DQS-LX	Anti-allergic eye drops		Epinastine 0.1%			Fluorometholone 0.1%	
27	DQS-LX	Anti-allergic eye drops			Olopatadine		Fluorometholone 0.1%	
28	DQS-LX						Fluorometholone 0.1%	IPL
29	DQS-LX	Anti-allergic eye drops		Epinastine 0.1%		Azithromycin		IPL
30	DQS-LX							
31	DQS-LX					Azithromycin	Fluorometholone 0.1%	IPL
32	DQS-LX							
33	DQS-LX	Anti-allergic eye drops		Epinastine 0.1%			Fluorometholone 0.1%	IPL
34	DQS-LX					Azithromycin		
35	DQS-LX						Fluorometholone 0.1%	
36	DQS-LX						Fluorometholone 0.1%	IPL
37	DQS-LX					Azithromycin	Fluorometholone 0.1%	IPL
38	DQS-LX							IPL
39	DQS-LX	Anti-allergic eye drops		Epinastine 0.1%		Azithromycin	Fluorometholone 0.1%	
40	DQS-LX					Azithromycin	Fluorometholone 0.1%	IPL
41	DQS-LX					Azithromycin	Fluorometholone 0.1%	
42	DQS-LX	Anti-allergic eye drops		Epinastine 0.1%			Fluorometholone 0.1%	IPL
43	DQS-LX	Anti-allergic eye drops		Epinastine 0.1%			Fluorometholone 0.1%	
44	DQS-LX					Azithromycin	Fluorometholone 0.1%	IPL
45	DQS-LX	Anti-allergic eye drops		Epinastine 0.1%			Fluorometholone 0.1%	IPL
46	DQS-LX	Anti-allergic eye drops		Epinastine 0.1%		Azithromycin	Fluorometholone 0.1%	IPL
47	DQS-LX						Fluorometholone 0.1%	
48	DQS-LX	Anti-allergic eye drops		Epinastine 0.1%		Azithromycin	Fluorometholone 0.1%	IPL
