# Supplementary material for: Tolerability of Diquas LX on tear film and meibomian glands findings in a real clinical scenario
Source: PLoS One. 2024 Sep 26;19(9):e0305020. doi: 10.1371/journal.pone.0305020 (PMC11426461; doi:10.1371/journal.pone.0305020)
Supplement: S3 Table — (PDF) [file pone.0305020.s003.pdf]

ID	DQS/DQS-LX group	SPEED	TMH	Plugging	Vascularity	FBUT	CFS	Presence of conjunctival hyperemia and papillae	Meibum grade	Meiboscore	Schirmer's test
1	DQS	8	0.23	0	0	4	0	Yes	0	0	5
2	DQS	6	0.17	3	2	3	0	Yes	2	3	7
3	DQS	8	0.16	3	0	4	0	Yes	2	1	4
4	DQS	7	0.22	2	0	4	0	Yes	2	2	5
5	DQS	7	0.24	1	0	2	0	Yes	1	2	5
6	DQS	6	0.16	2	0	5	1	No	1	2	1
7	DQS	8	0.24	3	2	4	0	No	2	2	3
8	DQS	7	0.23	2	2	3	0	Yes	2	2	4
9	DQS	6	0.18	2	2	4	0	Yes	1	4	13
10	DQS	6	0.37	2	2	4	0	Yes	2	2	25
11	DQS	6	0.29	2	3	5	0	No	1	4	6
12	DQS	8	0.3	2	2	3	0	Yes	1	3	6
13	DQS	11	0.25	3	3	2	0	Yes	2	4	1
14	DQS	7	0.26	2	1	3	1	No	1	4	8
15	DQS	5	0.26	2	2	3	1	No	1	4	2
16	DQS	23	0.18	1	0	1	0	Yes	0	2	6
17	DQS-LX	8	0.16	3	1	5	0	No	3	3	4
18	DQS-LX	15	0.3	3	0	4	0	No	3	1	4
19	DQS-LX	4	0.12	3	1	3	1	No	3	1	6
20	DQS-LX	11	0.18	3	1	3	2	No	2	2	1
21	DQS-LX	14	0.2	1	0	1	0	Yes	1	2	4
22	DQS-LX	15	0.14	2	0	2	4	No	1	2	0
23	DQS-LX	7	0.15	3	3	3	0	No	2	2	16
24	DQS-LX	28	0.13	2	1	1	0	No	2	4	5
25	DQS-LX	21	0.14	3	0	3	3	No	3	2	5
26	DQS-LX	7	0.18	2	1	4	1	Yes	2	2	1
27	DQS-LX	14	0.12	2	2	4	3	Yes	2	5	6
28	DQS-LX	8	0.17	3	3	2	6	No	2	5	0
29	DQS-LX	17	0.11	3	1	1	1	Yes	2	1	2
30	DQS-LX	7	0.12	3	2	5	1	No	2	2	5
31	DQS-LX	28	0.18	3	3	2	0	No	2	2	1
32	DQS-LX	16	0.15	1	0	1	0	No	3	2	13
33	DQS-LX	12	0.13	3	1	1	9	Yes	1	2	1
34	DQS-LX	10	0.18	3	1	2	0	No	2	3	6
35	DQS-LX	14	0.1	3	3	0	2	No	2	2	2
36	DQS-LX	16	0.09	2	1	4	2	No	2	2	5
37	DQS-LX	10	0.1	2	2	1	2	No	1	5	5
38	DQS-LX	28	0.12	2	2	1	5	No	2	5	4
39	DQS-LX	15	0.16	3	2	2	0	Yes	2	3	5
40	DQS-LX	20	0.12	3	2	4	0	No	2	3	1
41	DQS-LX	13	0.12	3	3	2	4	No	3	3	2
42	DQS-LX	28	0.17	3	3	1	6	Yes	2	4	3
43	DQS-LX	20	0.27	3	1	1	3	Yes	2	3	0
44	DQS-LX	21	0.18	3	2	1	0	No	2	3	8
45	DQS-LX	24	0.15	3	2	1	3	Yes	2	3	0
46	DQS-LX	20	0.17	3	2	1	0	Yes	1	3	1
47	DQS-LX	21	0.18	3	3	3	3	No	2	3	4
48	DQS-LX	24	0.11	3	3	1	4	Yes	2	3	3
