# Supplementary material for: Tolerability of Diquas LX on tear film and meibomian glands findings in a real clinical scenario
Source: PLoS One. 2024 Sep 26;19(9):e0305020. doi: 10.1371/journal.pone.0305020 (PMC11426461; doi:10.1371/journal.pone.0305020)
Supplement: S4 Table — (PDF) [file pone.0305020.s004.pdf]

ID	DQS/DQS-LX group	Eye stickiness in the morning	Increased eye discharge	Itciness after eye drops	Wanting to eye drops more than three times per day	Eye irritation
1	DQS			1		
2	DQS	1	1	1		1
3	DQS	1	1	1		
4	DQS	1	1	1		
5	DQS	1		1	1	
6	DQS	1	1		1	
7	DQS	1	1			
8	DQS	1	1		1	
9	DQS	1		1		
10	DQS	1	1	1		
11	DQS	1	1			
12	DQS	1				
13	DQS		1		1	
14	DQS		1			1
15	DQS	1	1			
16	DQS	1	1	1		1
17	DQS-LX					
18	DQS-LX					
19	DQS-LX					
20	DQS-LX					
21	DQS-LX					
22	DQS-LX					
23	DQS-LX					
24	DQS-LX					
25	DQS-LX					
26	DQS-LX					
27	DQS-LX					
28	DQS-LX					
29	DQS-LX					
30	DQS-LX					
31	DQS-LX					
32	DQS-LX					
33	DQS-LX					
34	DQS-LX					
35	DQS-LX					
36	DQS-LX					
37	DQS-LX					
38	DQS-LX					
39	DQS-LX					
40	DQS-LX					
41	DQS-LX					
42	DQS-LX					
43	DQS-LX					
44	DQS-LX					
45	DQS-LX					
46	DQS-LX					
47	DQS-LX					
48	DQS-LX					
